# Self-Powered Wireless Sensor Using a Pressure Fluctuation Energy Harvester

**DOI:** 10.3390/s21041546

**Published:** 2021-02-23

**Authors:** Jesus Javier Aranda, Sebastian Bader, Bengt Oelmann

**Affiliations:** Department of Electronics Design, Mid Sweden University, 85170 Sundsvall, Sweden; javier.aranda@miun.se (J.J.A.); bengt.oelmann@miun.se (B.O.)

**Keywords:** energy harvesting, piezoelectric energy harvesting, pressure fluctuation, self-powered sensor, wireless sensor nodes, integration with wireless sensors

## Abstract

Condition monitoring devices in hydraulic systems that use batteries or require wired infrastructure have drawbacks that affect their installation, maintenance costs, and deployment flexibility. Energy harvesting technologies can serve as an alternative power supply for system loads, eliminating batteries and wiring requirements. Despite the interest in pressure fluctuation energy harvesters, few studies consider end-to-end implementations, especially for cases with low-amplitude pressure fluctuations. This generates a research gap regarding the practical amount of energy available to the load under these conditions, as well as interface circuit requirements and techniques for efficient energy conversion. In this paper, we present a self-powered sensor that integrates an energy harvester and a wireless sensing system. The energy harvester converts pressure fluctuations in hydraulic systems into electrical energy using an acoustic resonator, a piezoelectric stack, and an interface circuit. The prototype wireless sensor consists of an industrial pressure sensor and a low-power Bluetooth System-on-chip that samples and wirelessly transmits pressure data. We present a subsystem analysis and a full system implementation that considers hydraulic systems with pressure fluctuation amplitudes of less than 1 bar and frequencies of less than 300 Hz. The study examines the frequency response of the energy harvester, the performance of the interface circuit, and the advantages of using an active power improvement unit adapted for piezoelectric stacks. We show that the interface circuit used improves the performance of the energy harvester compared to previous similar studies, showing more power generation compared to the standard interface. Experimental measurements show that the self-powered sensor system can start up by harvesting energy from pressure fluctuations with amplitudes starting at 0.2 bar at 200 Hz. It can also sample and transmit sensor data at a rate of 100 Hz at 0.7 bar at 200 Hz. The system is implemented with off-the-shelf circuits.

## 1. Introduction

Energy harvesting from pressure fluctuations in hydraulic systems can enable self-powered wireless sensors. Pressure fluctuations are a by-product of the system operation, often regarded as unwanted acoustic noise, and ubiquitous in the pressurized fluid. The abatement of this noise is mandatory on all hydraulic machinery since it can cause such problems as pipe leakage, the fatigue of mechanical components, and auditory damage [1,2,3]. Several studies have investigated pressure fluctuation energy harvesters (PFEHs) for converting this acoustic energy into electrical power to enable self-powered systems. These studies have mainly focused on the subsystems of the PFEH. For instance, the fluid-to-mechanical interface [4,5,6], performance improvement through acoustic resonance [7,8], and interface circuits [9] have been investigated. These investigations show that the adoption of pressure fluctuation energy harvesting, and consequently self-powered systems based on PFEHs, can lead to the elimination of unwanted wired connections and batteries, increasing the flexibility to deploy sensors and reduce maintenance costs.

Self-powered wireless sensors can have many applications in industrial contexts. For example, excavators, hoists, and other hydraulic machinery often undergo a hydraulic pressure change before a fault occurs. Often, a visual inspection is required to read analogue gauges at various locations on the machine [10]. This can lead to dangerous situations for personnel (e.g., high-pressure leaks), machine downtime, and time-consuming rounds of inspections. PFEHs can increase the deployment flexibility of wireless sensors and enable the measurement of pressure data without visual inspection. In addition, other applications such as hydraulic motors require temperature, flow, rotation, and pressure readings from the system. Wired connections and batteries are not an ideal solution for these types of motors, as maintenance or battery replacement can be difficult. Self-powered devices can transmit sensor data to the main system controller in the pump, where a Bluetooth Low Energy (BLE) central can be installed [11,12].

The development of PFEH has been of great interest over the last decade, with different harvesters and approaches being adapted to different hydraulic environments. Skow et al. [8,9,13] have reported on various PFEHs with multilayer piezoelectric stacks for low- and high-pressure systems. Their studies focused on the pressure fluctuation generated by a hydraulic pump with contributions in the range of 225 Hz–1000 Hz. Although their proposed harvesters report AC power up to 13 mW for a pressure amplitude of 2 bar, they have not shown any evaluation of the harvester in a fully integrated system. Another PFEH using a piezoelectric stack was reported by [14]. The PFEH was designed with an amplified stack structure operating in a water system with a static pressure of 2 bar. The authors reported simulated results with an open circuit voltage of 83.6 mV RMS at a pressure fluctuation with an amplitude of 0.095 bar and a frequency of 93.19 Hz. However, the study does not investigate a suitable power conditioning solution under these conditions. Ren et al. [15] reported an electromagnetic PFEH in a gas environment with pressure fluctuations of 0.4 bar at 20 Hz, showing 1.2 V and 2.0 mW of AC power at a resistive load. The system was designed for a static pressure of 1 bar. No power conditioning was specified. An alternative PFEH device using a flexible diaphragm with a piezoelectric was described in [16]. The authors reported 0.7 nW AC power for an excitation of 0.003 bar at 52 Hz, but no power conditioning or system implementation was discussed.

It is evident that, apart from the studies of Skow et al. the majority of studies on PFEH are tailored to very low static pressures in an attempt to design mechanical resonant mechanical structures. For higher static pressures, which are more common in industrial hydraulic systems, resonant structures are not practical as they may fail due to the high stresses [4,8,16,17]. Compared with the other PFEH designs, the advantages of the PFEH with multilayer piezoelectric stack, as reported in [4,7,8,14], are the higher static pressure rating and the advantages of the stack for off-resonance energy generation.

Although the literature shows that the development of PFEH is of interest, there are few studies that address end-to-end implementation, i.e., the development of self-powered devices with PFEH. Indeed, studies of self-powered sensor systems operating via energy harvesting of pressure fluctuations are currently limited to proof-of-concept implementations at single operating points. In [12], Schwartz et al. evaluated a full system implementation. They investigated the output power of a PFEH and measured the power consumption of a commercial wireless sensor system, as well as the feasibility of energy-autonomous operation. Their study focused on the operating conditions of an industrial pump with pressure fluctuations at 225, 450 and 675 Hz. The system was optimized for a dominant pressure fluctuation frequency at 450 Hz with an amplitude of about 2 bar. At this excitation, their PFEH implementation produced an output power of 2.6 mW. Toothman et al. [11] studied a different wireless sensing system under the same operating conditions. Their work assumed the same output power as determined by Schwartz et al. and theoretically evaluated the achievable performance of the sensing system. Consequently, their study was limited to the same excitation condition, and losses in the interface circuits were overlooked.

To support the use of PFEH in a wider range of operating conditions, various methods and techniques such as (a) fluid-mechanics interface optimization [6], (b) acoustic impedance matching [7,8], (c) circuit techniques that enable efficient rectification at low input voltage [9,18,19], and (d) circuit techniques to increase power generation can be used to enhance the performance of the PFEH [20,21,22]. The focus of this work is to propose and evaluate a self-powered wireless pressure sensor (SP-WPS) considering pressure fluctuations with amplitudes less than 1 bar at frequencies less than 300 Hz. Systems with lower acoustic power density (lower frequencies and amplitudes) are common in a variety of industrial applications [7,9,14], and the lower power density makes obtaining sufficient energy challenging.

First, we measure the power requirements of the wireless sensor, which consists of a pressure sensor and a BLE-enabled System on Chip (SoC), and highlight the design tradeoffs for its implementation. Second, we perform an experimental analysis of the frequency response of the PFEH and show the estimated power and the impact of a resonator on power generation. Third, we integrate and evaluate the complete system to show the conditions for system start-up and operation, namely the minimum pressure amplitude and frequency, and the overall performance of the prototype. In addition, we present an analysis of the performance enhancement using a power improvement unit. The study presents the implementation of a self-powered synchronized switch on inductor circuit to improve the power generation performance of the PFEH. Compared to previously reported self-powered active power improvement units that require integrated circuit design, complex control techniques [23,24,25,26] or high-voltage amplitudes for operation [21,22,27,28], we present a circuit implementation adapted for lower voltages and using off-the-shelf components.

## 2. System Design and Implementation

**Figure 1:**
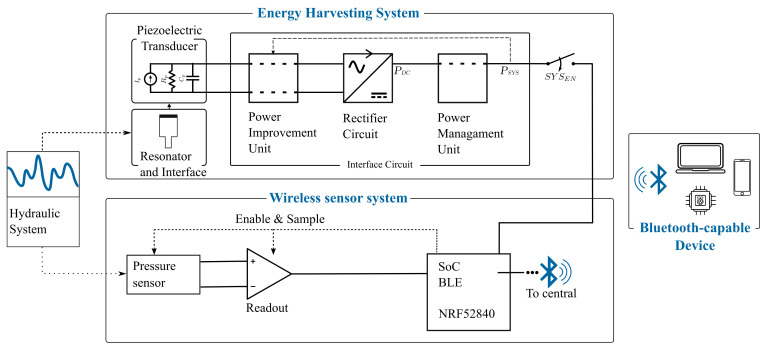
Overview of the self-powered wireless sensor and its subsystems.

Figure 1 shows the SP-WPS and its subsystems, namely the Energy Harvesting System and the Wireless Sensor System (WSS). The energy harvesting system consists of (a) a PFEH to convert pressure fluctuations in hydraulic systems into AC power and (b) an interface circuit to convert AC power into DC power. The wireless sensor system has two main components: (a) a hydraulic pressure sensor and (b) a SoC with BLE capabilities that controls the acquisition, processing, and transmission of data. The pressure data transmitted by the SoC can be read by any BLE-enabled receiver. The SP-WPS is designed to be active only when the hydraulic system is operating, enabled by the $SYS_{EN}$ signal. Pressure fluctuations are a form of noise in hydraulic systems that are constantly present when the main energy source, i.e., the pump, is in operation [1,2,3]. Long-term energy storage is therefore not included in the proposed system implementation, but it can be added for other application scenarios if required.

**Figure 2:**
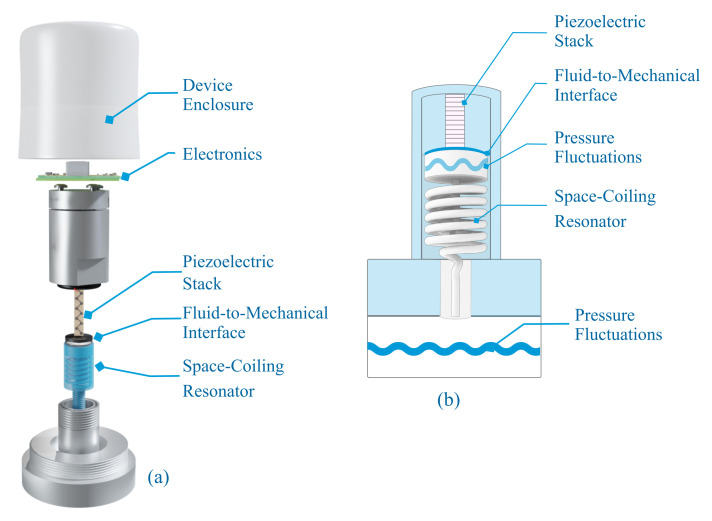
(**a**) Overview of the self-powered wireless sensor detailing the different components. (**b**) General principle of the pressure fluctuation energy harvester and its components.

Figure 2 shows the different components of the physical SP-WPS prototype and the general working principle of the PFEH. The PFEH can be connected to any port in a hydraulic system where there is access to hydraulic fluid.

### 2.1. Pressure Fluctuation Energy Harvester

The PFEH converts pressure fluctuations into electrical energy by converting dynamic pressure fluctuations. The characteristics of the pressure fluctuations are directly related to the operating variables and the design of the pump. Previous works associate the frequency and amplitude of pressure fluctuations with the static pressure and speed of the main pump in a system [1,2,3,4,7]. In these works, it was reported that the amplitude of the pressure variation can reach 10% of the static operating pressure.

Considering that the dynamic pressure fluctuations are only a fraction of the static pressure, the PFEH structure must be able to withstand high-pressure forces and still capture the small dynamic pressure fluctuations. In these scenarios, resonant energy harvesters, such as cantilevers, cannot be used [4,23,29,30]. For this reason, a multilayer stack of lead zirconate titanate (PZT) is used in this work. This transducer has shown high compressive strength, superior off-resonance performance, and higher static capacitance compared to other transducers [4,31]. The PZT stack converts mechanical force into electrical energy through the direct piezoelectric effect. The open circuit voltage generated is given by

(1) $$V_{p}=\frac{d_{33}^{*}Fsin\left(\omega t\right)}{C_{p}}.$$

where $d_{33}^{*}$ is the effective piezoelectric coefficient, *F* is the force amplitude, and $C_{p}$ is the capacitance of the piezoelectric stack [31]. The force compressing the PZT stack results from the pressure fluctuations acting on the fluid-to-mechanical interface, a thin metal plate connecting the stack to the fluid, as shown in Figure 2.

The fluid-to-mechanical interface converts pressure fluctuations into axial mechanical force and protects the stack from the fluid. The interface is installed between two stainless steel housings. Both the static pressure and the amplitude of the pressure fluctuations are considered in the design of the fluid-mechanical interface. The diameter and thickness of the plate are based on the operating characteristics of the hydraulic system. A larger diameter may result in higher power generation, but it may compromise the integrity of the PZT by increasing the static pressure force. The force transmission ratio describes the ratio of pressure to force that a fluid interface can achieve. In [4,5,6], the authors reported pressure-to-force transfer ratios ranging from 0.5 to 0.9, depending on the design, material, and dimensions of the fluid-mechanical interface.

Recent studies have shown that adding an acoustic resonator or compliant structure can significantly increase the power generation of an acoustic energy harvester [32]. In hydraulic systems where compliant systems are not feasible due to high static pressure [7,8,32], acoustic resonators can increase power generation without increasing the static load on the transducers. For example, Helmholtz Resonator, have shown pressure fluctuation amplification for frequencies greater than 450 Hz [8] and SCR [7] with good amplification performance in the range of 200 Hz to 1000 Hz. The gain is a function of the design material, volume, frequency, and hydraulic fluid properties [7]. Space-coiled structures are of great interest for hydraulic applications and energy harvesting of acoustic noise in air due to their improved acoustic amplification performance [7,32,33,34]. In this study, a SCR is used to improve the performance of the PFEH for frequencies below 300 Hz to avoid excessive static compressive forces on the transducer (without increasing the fluid-to-mechanical interface area). The design of the SCR is made possible by 3D printing techniques that allow the design and fabrication of more complex geometries [7]. The SCR is an acoustic resonator based on the classical Helmholtz resonator. Compared to the Helmholtz Resonator, the SCR can provide better performance at lower frequencies with the same volume constraints, i.e., it can resonate at lower frequencies without increasing the volume of the resonator. Essentially, the SCR benefits from a long neck and wider neck opening, which improves acoustic gain due to lower narrow-neck losses and enables the design of compact structures [7,8,32]. The SCR allows the transmission of pressure fluctuations from the fluid in the hydraulic system to a small chamber where the interface between the fluid and the stack is installed. Figure 2b shows the main working principle of the PFEH and the SCR.

The theoretical effective AC power that can be generated by the PFEH without interface circuit, can be estimated by

(2) $$P_{AC}=\frac{\pi fC_{p}V_{p}^{2}}{\sqrt{2}}.$$

According to [31], up to 70% of effective AC power can be delivered to a matched resistive load without power improvement circuits. However, in a real system, the DC power that can be delivered to a load depends on the performance of the PFEH in combination with the interface circuitry, i.e., the performance of the rectifier, the power improvement circuit, and the efficiency of the power management unit (PMU).

### 2.2. Interface Circuit

**Figure 3:**
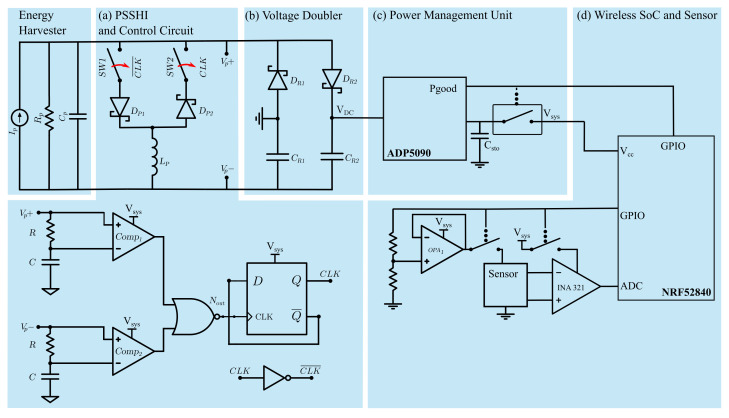
Schematic of the self-powered wireless pressure sensor system.

Since embedded systems cannot employ AC power to operate, proper power rectification and conditioning is required. The interface circuit rectifies, stores, and manages the AC power generated by the piezoelectric transducer. As depicted in Figure 1, the interface circuit comprises a power improvement unit (PIU), a rectifier circuit, and a PMU. Figure 3 shows a schematic of the circuit implementation in this work. The circuit consists mainly of (a) Parallel Synchronized Switch Harvesting in Inductor (PSSHI) as a power improvement unit; (b) voltage doubler for rectification; (c) PMU with system capacitors and load switch to disconnected the system load from the interface circuit; and (d) wireless SoC (NRF52840) and sensor system.

Since no long-term energy storage is used in this implementation, the interface circuit must convert enough AC to DC power to meet the minimum requirements for self-powered operation. This limitation is mainly dictated by the PMU, which requires a minimum DC voltage ($V_{DC}^{cs}$) and power ($P_{DC}^{cs}$) to start. The system load, i.e., the wireless sensor system, does not consume power until the PMU reaches a predetermined voltage.

A voltage doubler is used for rectification. This topology facilitates the start of the PMU by providing a step-up to reach the minimum $V_{DC}^{cs}$. Furthermore, preliminary work shows the improvement of the voltage doubler over other passive topologies such as the full-wave bridge rectifier [35]. In addition, the voltage doubler enables the use of the PSSHI as PIU. The PSSHI is a circuit technique that increases power by inverting the voltage across the piezoelectric transducer at each voltage peak by inducing a resonant circuit formed by the internal capacitance of the PZT stack ($C_{p}$) and an external inductor ($L_{p}$). In this way, the circuit can harvest the energy that would otherwise be lost when charging the internal capacitance of the piezoelectric [20,23,24,36]. Voltage inversion is achieved by two analog switches (SW1 and SW2) that are activated at each voltage peak. The switches are controlled by two peak detectors implemented with low-power comparators and simple logic gates, as shown in Figure 3.

The low-power comparators ($Comp_{1}$ and $Comp_{2}$) detect the difference between the voltage $V_{p}+$ and $V_{p}-$ and the signal modified by the RC circuit. The $N_{out}$ signal drives a frequency divider that generates the clock signal to control the analog switches of the PSSHI. When one of the switches is closed, a resonant network is created between the stack ($C_{p}$) and the inductor ($L_{p}$), causing a voltage inversion. After the inversion, the reverse current is stopped by the diode ($D_{p1}$ or $D_{p2}$) [27]. In practice, the voltage inversion efficiency and power generation improvement is a function of frequency, open circuit voltage, diode voltage drop, and peak detection accuracy [20,27,35,37]. This circuit implementation is a simple control technique that can improve the power generation of the PFEH. However, it deviates from the ideal PSSHI technique due to the voltage drop of the diode in the inversion network and the delay introduced by the peak detection implementation.

**Figure 4:**
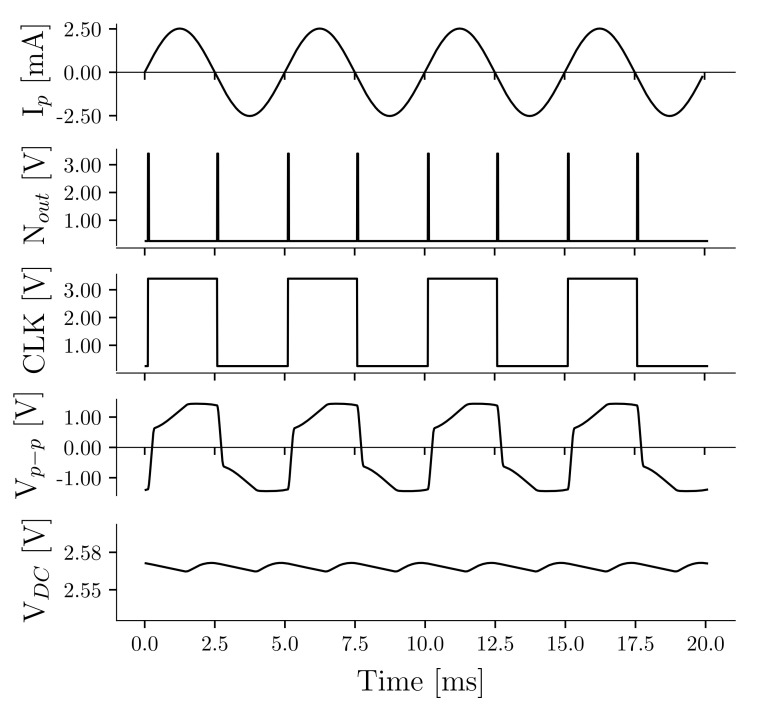
Waveforms of the voltage doubler and parallel synchronized switch on inductor during operation at 200 Hz.

Figure 4 shows the waveforms for the operation of the voltage doubler in combination with the PSSHI. The operating frequency is 200 Hz with an open circuit voltage $V_{p}\approx$ 0.7 V. The figures show the estimated current generated by the stack due to the pressure fluctuations, the output of the logic gate ($N_{out}$), the clock signal (CLK), the voltage across the stack $V_{p-p}$, and the output voltage ($V_{DC}$) supplied to the power management unit.

We have implemented the PSSHI circuit using low-power, off-the-shelf components, which enables low-power design and implementation in low-volume applications. In this implementation, low impedance analog switches (Texas Instruments TS3A4741) and a passive inductor are used. The diodes used are the CTS05S30. Compared to previously reported circuit implementations of the PSSHI for stack-based energy harvesters (e.g., [22]), we have focused particularly on a design that operates at low voltages. The PSSHI is an active, self-powered PIU, which starts operating after the PMU is fully started. Figure 3 shows the voltage doubler, the PSSHI circuit implementation, and the control circuit. Previous implementations of the PSSHI have focused on harvesting vibrational energy using cantilevers, which are typically operated at resonance. These types of transducers generally produce large open circuit voltage amplitudes. Common implementations of PSSHI control circuits for these cases consist of digital controls that can be very power hungry (up to 1 mW) [26]; bipolar transistor switches which require large voltage amplitudes for operation (typically > 1 V) [20,22,38]; or integrated circuits that, despite their low input voltage requirements, can become costly for low-volume applications.

Other studies suggest the use of complex impedance matching for PZT stacks [13]. However, for frequencies below 300 Hz, the required size of the matching inductance may be impractically large. For example, a PZT stack with a capacitance of 3 μF at an excitation frequency of 200 Hz would require an inductor of about 0.2 H. The PSSHI typically requires smaller inductances [20,25,35], e.g., less than 10 mH for the same operating conditions. Table 1 shows the components for the PSSHI implementation and their power consumption, demonstrating that a low-power implementation can be achieved even with commercially available components.

As a PMU, the proposed SP-WPS uses an ADP5090 by Analog Devices, an integrated boost regulator with maximum power point tracking (MPPT) to improve power transfer. It also provides a *Power-good* ($P_{good}$) terminal that informs when sufficient energy is available for stable operation and prevents the load from drawing current before this happens. The commercial PMU is a pragmatic alternative to other implementations consisting of custom voltage regulators, maximum power point tracking circuits, energy aware interfaces, and integrated circuit design [19,23,39,40]. In this implementation, the output of the voltage doubler ($P_{DC}$) supplies power to the PMU. The first phase of operation is to charge the storage capacitor of the PMU (cold start). During this phase, the PMU regulates the voltage to $V_{DC}^{cs}$ until the capacitor exceeds a preprogrammed voltage threshold. When the storage capacitor ($C_{sto}$) is charged, the PMU enables $P_{good}$ to activate the main system switch (Texas Instruments TPS22860), which starts the operation of the SoC and the PIU. The second phase of operation consists of the sampling and transmission of pressure data processed by the SoC. During this phase, the PMU continues to charge the capacitors and supply power to the load. If the harvested energy exceeds the energy required by the load, the PMU turns off the input to avoid overcharging. In addition, during this phase, the PMU activates the maximum power point tracker to match the source (PFEH and voltage doubler) and optimize power transfer. Due to the low input voltage requirements of $V_{DC}^{cs}=$ 380 mV and the minimum power of 16 μW, the ADP5090 is suitable for this implementation.

In this study, in order to consider the influence of the rectifier and the efficiency of the PMU in a single figure of merit (FOM), we analyze the AC-DC conversion performance of the whole interface circuit as the ratio of the power that can be delivered to the system to the effective AC power that can be generated, as

(3) $$FOM=\frac{P_{sys}}{P_{AC}}.$$

### 2.3. Wireless Sensor

The wireless sensor consists of an industrial pressure sensor and a SoC with BLE communication capabilities. The pressure sensor is a Wheatstone bridge ( 350 Ω) with a nominal excitation voltage of 5 V. The low impedance of the sensor makes low-power design difficult. Nevertheless, low-power bridge conditioning circuits are becoming increasingly common. Common design strategies include lowering the excitation voltage or strobing the drive power [41]. In this work, a voltage divider is used to select the excitation voltage of the sensor ($V_{ref}$), and a low-power instrumentation amplifier (INA321) is used for signal conditioning. With this configuration, the estimated power requirement of the sensor is 2.85 mW/V^2^. A lower excitation voltage and fast sampling can result in low power consumption. The tradeoff is to reduce the output signal of the bridge. The reduced drive voltage produces a correspondingly reduced bridge signal, reducing the signal-to-noise ratio [41]. The sensing performance tradeoff is application dependent, and software and circuit strategies can further optimize low-power operation [42,43,44].

The WSS uses the NRF52840 SoC to activate, read, and transmit pressure sensor data at fixed intervals via non-connectable advertisement packages (advertising-only mode) and characteristic notification packets (connectable mode). Figure 3 shows the schematic configuration of the WSS and components. With this configuration, any BLE-enabled device can receive the pressure data and perform additional operations or upload the information to a server. After system initialization (first phase of operation), the SoC starts sampling and reading the sensor data at a fixed interval. For connected mode, the SoC begins sampling and sending pressure data after the system connects to a BLE-enabled device. The NRF52840 processes these events in a sequence with predefined timers. First, the NRF52840 wakes up at a predefined time before a radio event and activates the sensor and its conditioning circuitry. After a small delay, the ADC samples and processes the data. After the data transmission, the NRF52840 goes back to idle-mode.

## 3. Methods

### 3.1. Experimental Setup and Prototype

**Figure 5:**
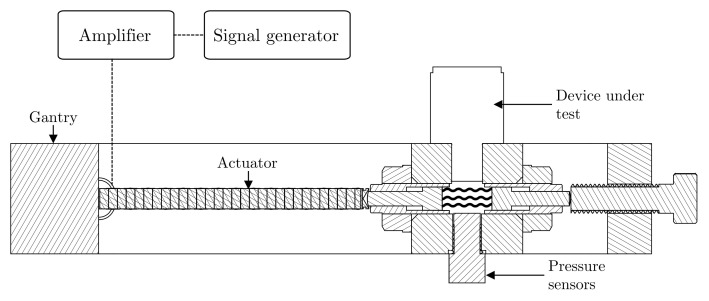
Apparatus for the exploration of the pressure fluctuation energy harvester [45].

In order to evaluate the PFEH in a controlled environment and investigate its response to different excitation characteristics, this study employs an apparatus that can reproduce the working conditions of a hydraulic system. The experimental setup can generate custom pressure fluctuation signals in a small volume of fluid (ISO VG-32) with a linear actuator. Pressure fluctuations can be generated with frequencies up to kHz, and static pressure can be set manually. Pressure is monitored by pressure sensors (PCB 113B26, Honeywell model S). Figure 5 shows the diagram of the apparatus used in this work, and more details about it can be found in [45].

**Figure 6:**
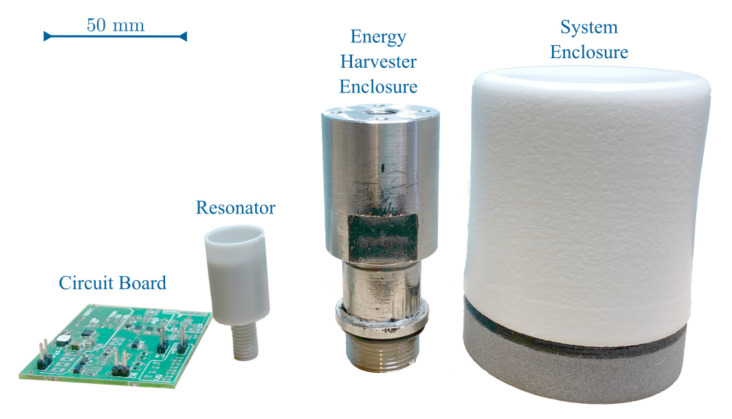
Picture of the system and its components.

The PFEH prototype consists of a 3D-printed nylon SCR, a commercial multi-layer piezoelectric stack, and a fluid-to-mechanical interface. The resonator and stack are enclosed in a metal housing as shown in Figure 2. The properties of the multi-layer piezoelectric stack (ThorLabs PK4FYP2) used in the PFEH are shown in Table 2. The fluid-to-mechanical interface is a stainless steel plate with a diameter of 16 mm and a thickness of 50 μm. Figure 6 shows a picture of the prototype.

### 3.2. System Evaluation

The evaluation of the SP-WPS consists of three analyses. In the first analysis, we experimentally measure the power consumption of the WSS using a Power Analyzer N6705C and an MSOX3024T oscilloscope during sensor sampling, wireless transmission, and *idle-mode* for a $V_{sys}=$ 3.3 V. This analysis allows the elaboration of a power profile of the WSS operation.

In the second analysis, the frequency response of the PFEH is studied for a swept sinusoidal pressure signal in the range of 25 Hz to 300 Hz. The static pressure is set to 35 bar. We measure the piezoelectric voltage $V_{p}$ with an oscilloscope and calculate the theoretical effective AC power that can be obtained at each frequency using (2). This measurement shows the influence of the resonator and the estimated harvested AC power given the pressure fluctuation amplitude.

The third analysis evaluates the performance of the complete SP-WPS for pressure fluctuations with amplitudes in the range of 0.2 bar to 0.8 bar at 200 Hz. These characteristics correspond to the expected operating environment of a commercial hydraulic motor with low-speed and high-torque [7]. We measure the power at the PMU ($P_{sys}$) and, based on the energy demand of the WSS measured in the first analysis, estimate the achievable sampling rate of the system. In addition, we calculate the FOM describing the performance of the system to show the difference between power generation with and without PIU for a range of excitations.

## 4. Results and Discussions

### 4.1. Wireless Sensor Power Consumption

**Figure 7:**
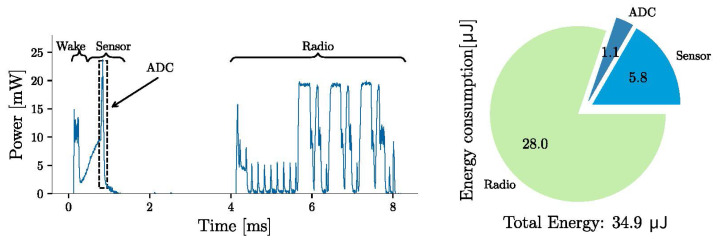
Power profile for the sensor sampling and BLE communication for the advertising-only mode. The calculated energy is for one cycle of operation.

**Figure 8:**
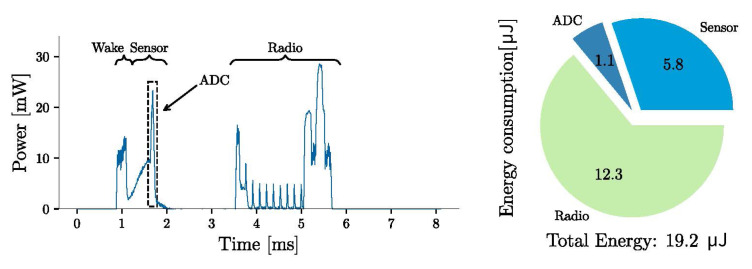
Power profile for the sensor sampling and BLE communication for the connectable mode. The calculated energy is for one cycle of operation.

Figure 7 and Figure 8 show the power profiles of the wireless sensor for the “advertising-only” and “connectable” modes, respectively. The energy consumption in the figures are the energy costs for performing the task once (in one cycle of operation). The power profiles show the different events for an operating voltage of 3.3 V. The system in idle mode consumes on average 13 μW. The energy required for sampling and data processing is 6.9 μJ, which is about one-fourth of the energy required for data transmission in advertising-only mode. For the connectable mode, the energy required for data transmission ( 12.3 μJ) is comparable to the energy required for sensor sampling, and less than half of the energy required for radio transmission in advertising-only mode ( 28 μJ). The SoC limits the sampling rate to about 8 Hz for the advertising-only mode and 100 Hz for the connectable mode. So, the connectable mode has lower power consumption and faster sampling rates. However, the energy required to connect the SoC to a BLE-enabled central device can be up to a few milli-joules, resulting in a significant startup delay. Advertising-only mode does not require a connection event. In this mode, the system can start sampling and sending sensor data without waiting for a BLE-enabled device to connect.

The measurements consider the excitation voltage of the sensor ($V_{ref}$) at 1 V, which has been selected based on a trade-off between sensing accuracy and energy consumption. As was previously discussed, there exist different software and hardware techniques, such as oversampling and noise shaping [42], power strobing [41], sampling and hold and turn on optimization [43], and custom interfaces for Wheatstone-bridge based sensors [44] that can overcome the challenges of low-energy sensing.

### 4.2. Frequency Response and Power Estimation

**Figure 9:**
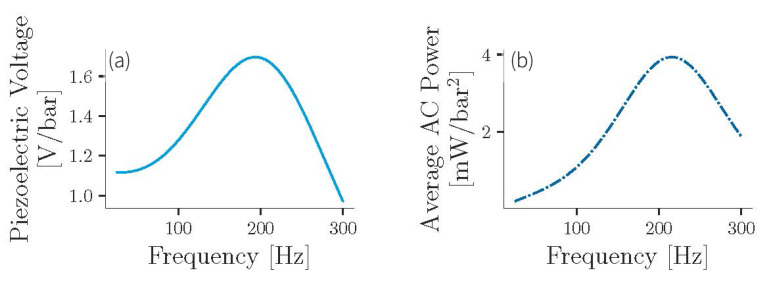
(**a**) Frequency response of the system. (**b**) Estimated average AC power as a function of pressure amplitude.

Figure 9 shows the frequency response detailing the measured open circuit voltage ($V_{p}$) and the calculated AC power ($P_{AC}$) as a function of the pressure amplitude obtained from the PFEH under sinusoidal excitation. The SCR shows resonance at about 200 Hz, increasing both the open-circuit voltage and $P_{AC}$. Indeed, considerable gain is observed in the 125 Hz to 250 Hz region. Thus, the SCR can enhance the power generation over a relatively wide bandwidth and in different scenarios. Due to the high resonant frequency of the piezoelectric stack of about 34 kHz, without the SCR the PFEH would have a constant open-circuit voltage even at higher frequencies, and the power would only increase linearly with frequency [7,31].

Figure 9 suggests that a pressure amplitude of 0.1 bar is sufficient to generate enough power to start the PMU. However, the AC-DC conversion involves losses and minimum operating requirements that may affect system performance. For full system integration, it must be taken into account that the PFEH in combination with the rectifier has a different power optimum at different excitation. Therefore, the PMU must utilize the MPPT to improve power transfer and provide sufficient power to the load for continuous operation.

### 4.3. Full System Implementation Analysis

Table 3 shows the comparison between the characteristics of the interface circuit used in our SP-WPS implementation and other relevant studies, both with piezoelectric cantilevers and stacks. A commonly used metric to compare the performance of the interface circuit for piezoelectric energy harvesters is to obtain the ratio of DC power ($P_{sys}$) obtained after the interface circuit and the power that can be obtained with an ideal full-wave bridge rectifier (improvement = $P_{sys}{\left(C_{p}fV_{p}^{2}\right)}^{-1}$) [25,46]. For the cases of cantilever energy harvesters using PSSHI, the most common approaches for self-powered operation are to use switches based on bipolar junction transistors (BJT) or integrated circuits. BJT switches are constrained to a minimum input voltage for operation, i.e., the voltage generated by the transducer must be greater than the forward voltage of the diodes and the saturation voltage of the transistors in the inversion network. Even for optimized structures that have demonstrated operation at $V_{p}>1V$, their performance is poor at low input voltages [20,21,22,38]. PSSHI with digital controls and integrated circuit solutions have also been reported for piezoelectric cantilevers and generally show the best improvement. Although studies show PSSHI operation at very low input voltages with good performance (up to 4.75 times compared to the full-wave bridge rectifier) [19], there are no reports of circuits for piezoelectric stacks. In addition, integrated circuit design for low-volume implementations can be costly. For piezoelectric stacks, Liu et-al. [22] reported a factor of around 1.7 improvement for low frequencies (1.5 Hz to 2.3 Hz) and large input voltages (around 20 V) using a PSSHI with BJT switches. Skow et al. reported a voltage multiplier with impedance matching (additional indutance) showing a 4.8-fold improvement. However, for systems with lower frequencies of pressure fluctuations, impedance matching may not be feasible due to the size of the inductor required [13]. The table shows that, compared to the other PSSHIs for piezoelectric stacks operating outside resonance, our proposed interface circuit has comparable performance, requires only single-supply voltage comparators, and can operate at a lower $V_{p}$.

### 4.4. Evaluation of the Self-Powered Sensor System

**Figure 10:**
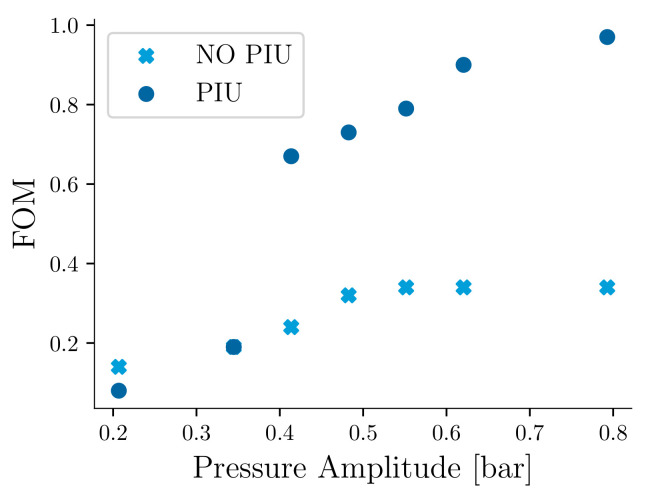
Performance of the system showing the figure of metric for the AC-DC conversion and the impact of the power improvement unit.

To investigate the impact of available power on WSS operation, we calculate the achievable sampling rates as a function of the pressure fluctuation amplitude. This study considers the entire system, including the harvester, interface circuit, and load. First, we calculate the *FOM* according to Equation (3) for the system with and without PIU, and the results are shown in Figure 10. For the lower range of pressure fluctuation amplitudes, the performance metric is less than 0.2, showing that a considerable amount of energy is lost in rectification and voltage boosting. For larger pressure fluctuation amplitudes, for the scenario without PIU, the *FOM* settles at about 0.33, showing the difficulty of efficiently extracting energy from the transducer without PIU. With PIU, the power increases significantly with increasing pressure fluctuation amplitude, reaching a *FOM* of 1 at about 0.8 bar. However, the performance at lower pressure amplitude (<0.4 bar) is limited and even drops below the performance without PIU.

**Figure 11:**
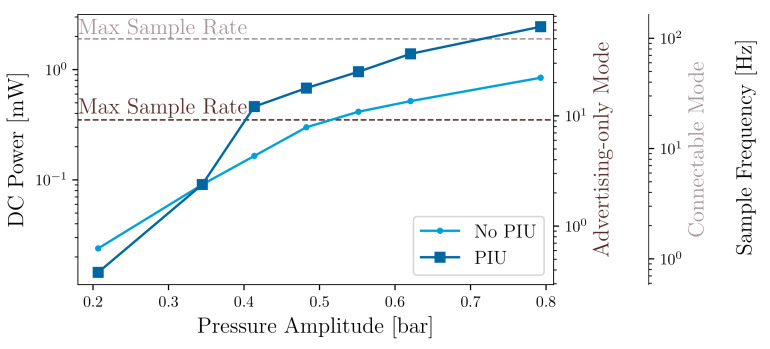
Measured DC power of the system and estimated sampling frequency.

Figure 11 shows the measured DC power $P_{sys}$ of the overall system at increasing pressure fluctuation amplitudes for a frequency of 200 Hz, as well as the calculated achievable sampling rate with the current program implementation. The figure shows that the system starts with as low as 0.2 bar ($V_{p}\approx$ 0.35 V), with an achievable sampling rate of approximately 0.5 Hz for advertising-only mode and 1.2 Hz for the connectable mode. The PIU impact is notable from 0.35 bar ($V_{p}\approx$ 0.6 V) and shows significant improvement at increasing amplitudes compared to the case without PIU. It achieves up to 3-fold improvement in the range of higher pressure fluctuation amplitudes. At about 0.4 ($V_{p}\approx$ 0.68 V), the system exceeds the required power to reach the maximum sampling rate for the advertising-only mode, which means that the system can allow higher sensor excitation, more sensor samples, or higher radio transmission power as amplitudes continue to increase. For pressure fluctuations with an amplitude of about 0.7 bar ($V_{p}\approx$ 1.2 V), the system can sample and transmit pressure data at a rate of nearly 100 Hz, the maximum sampling rate for the connectable mode. Further optimization of the PMU and PSSHI, particularly in the lower range of excitation, can improve overall performance and enable higher sampling rates. Table 4 shows a comparison of the performance and operating range of the proposed self-powered wireless sensor and the results of previous proposed solutions [11,12].

The PFEH performed well in the scenario studied, but some limitations should be noted. First, the PFEH was investigated with harmonic pressure signals. In real hydraulic environments, the pressure fluctuations may have more frequency components and vary in frequency over time [1,2]. This can lead to challenging scenarios, for example: (a) the resonant frequency of the SCR is fixed by design, limiting the operation of the PFEH to the gain band offered by the resonator. Changes in the frequency of pressure fluctuations can drop the performance of the PFEH and thus the impact of the sensor’s predefined transmission interval, and (b) the performance of the PSSHI may be affected by noise and other frequency contributions. Therefore, the next studies may address software or hardware implementations such as those presented in [39] to adjust the sensor transmission interval depending on the harvested energy levels. Moreover, the control circuit of the PSSHI and the power management circuit can be optimized to automatically adapt to the environment. Second, the system is designed for static pressures of less than 50 bar. Higher static pressures can damage the interface and piezoelectric transducer. For higher static pressures, the size of the fluid-to-mechanical interface and transducer should be revised to withstand higher compression forces.

## 5. Conclusions

This paper presented the design, development, and evaluation of a self-powered wireless pressure sensor, focusing on low amplitude and frequency excitation. We calculated the energy required for sampling and wireless data advertising of a commercial pressure sensor and achieved an energy cost of 34.9 μJ and 19.2 μJ per sample for the advertising-only and connected modes, respectively. Experimental measurements of the prototype implementation show that the self-powered system can start with as low as 0.2 bar ($V_{p}\approx$ 0.35 V) of pressure amplitude at a frequency of 200 Hz. The system has its highest power output at the resonance of the acoustic resonator used by the harvesting device, which is approximately 200 Hz. At this frequency, the pressure fluctuation energy harvester generates enough energy to provide the maximum sampling rate of about 100 Hz for a system with pressure fluctuations having an amplitude of about 0.7 bar ($V_{p}\approx$ 1.2 V). The self-powered wireless pressure sensor is designed assuming a standard hydraulic environment where pressure fluctuations are present whenever the host system is operating, and is not intended for intermittent energy harvesting that requires long-term energy buffering.

The system evaluation showed improvement in power generation, even at low excitation, with a parallel switch on inductor power improvement circuit employing only off-the-shelf circuits. The simple design of the power improvement unit allows its optimization for different operating ranges and a simple implementation. However, experimental measurements show that the PIU does not provide performance improvement for pressure fluctuations with amplitudes smaller than 0.35 bar and may even reduce the performance below this point. The operating range of the PSSHI is limited by the voltage drop of the diodes in the inversion network and the performance of the circuit that generates the control signals.

Self-powered wireless devices can minimize the use of wired connections, prevent maintenance in high-risk environments, and reduce operational costs. Taken together, the results show that our proposed system design succeeds in extending the operating range of current power conditioning circuits for pressure fluctuation energy harvesters to low excitation applications, thus filling the identified research gap. We have highlighted the design requirements and end-to-end implementation challenges. For example, the role of the space coiling resonator in power enhancement; the advantages of combining a voltage doubler and power improvement circuit to enable operation at low input excitation; and the feasibility of an interface circuit including a commercial power management unit for cold start, voltage regulation, and management of power generated by the pressure fluctuation energy harvester. Our study demonstrates the practicality of self-powered wireless sensors for condition monitoring in hydraulic applications and provides a framework that can be used as a basis for developing these devices.

## Figures and Tables

**Table 1:** Components for the parallel synchronized switch on inductor circuit.

Component	Voltage [V]	Quiescent Current [nA]
Comparator (TLV3691)	0.9–6.5	75
Analog switch (TS3A474X)	1.8–3.6	75
D Flip-Flop (SN74AUP1G74)	1.8–3.3	500
Logic Gates (NOR, NOT)	0.8–3.3	500

**Table 2:** Properties of PK4FYP2 multilayer piezoelectric stack.

Property	Value
$C_{p}$	3.5 ± %15 μF
$d_{33}^{*}$	≈240 nC/N^−1^
Resonant Frequency	34 kHz

**Table 3:** Interface circuit performance comparison to other relevant studies.

Reference	[22]	[19]	[9]	[26]	[47]	[18]	[35]	This Work
**Transducer**	Amplified Stack	MEMS	Stack	Stack	Cantilever	Cantilever	Cantilever	Stack
**Capacitance**	2.1 uF	2 nF	1.9 uF	661 nF	18 nF	30 nF	110 nF	3 uF
**Technique**	PSSHI	SSH	Impedance matching	PSSHI	PSSHI	PSSHI	PSSHI	PSSHI
**Control Circuit**	BJT switch	Digital	-	Digital	Comparator	Mosfet	Comparator	Comparator
**Type of Implementation**	Discrete	CMOS 180 nm	Discrete	Digital	Discrete	Discrete	Off-the-shelf	Off-the-shelf
**Open Circuit Voltage (*V_p_*)**	20 V	1.12 V	0.27 V	-	2.4 V	0.28–0.7 V	5 V	0.6–1.36 V
**Frequency**	1.5 Hz–2.3 Hz	317 Hz	450 Hz	18.7 Hz	225 Hz	100 Hz	112 Hz	200 Hz
****Self-Powered****	Yes	Yes	-	Yes	Yes	Yes	No	Yes
**Improvement** $P_{\mathrm{sys}}{\left({\mathrm{fC}}_{p}V_{p}^{2}\right)}^{-1}$	1.7	4.75	4.8	-	5.8	-	1.2	1–2
**Details**	-	-	Multiplier	-	-	-	-	-
**Operation Mode**	Off-resonance	Resonance	Off-resonance	Resonance	Resonance	Resonance	Resonance	Off-resonance
**DC-DC or Power Management**	No	Yes	No	Yes	No	No	No	Yes

**Table 4:** Comparison of self-powered wireless sensors for hydraulic systems.

Source	Minimum Pressure [bar]	Harvested Power [mW]	Operating Frequency [Hz]	Sensors	Sampling Rate [Hz]	Wireless Technology
[11]	2	2.6	450	Pressure and temperature	-	BLE
[12]	2	2.6	450	Pressure and temperature	333	2.4 GHz ISM
This Work	0.2	0.02–2.4	200	Pressure	up to 8:Advertising-Only up to 100:connected	BLE
